# Early warning and response system for dengue outbreaks: Moving from research to operational implementation in Mexico

**DOI:** 10.1371/journal.pgph.0001691

**Published:** 2023-09-20

**Authors:** Gustavo Sanchez Tejeda, David Benitez Valladares, Fabian Correa Morales, Jacqueline Toledo Cisneros, Brisia E. Espinoza Tamarindo, Laith Hussain-Alkhateeb, Corinne S. Merle, Axel Kroeger

**Affiliations:** 1 Formerly CENAPRECE (Centro Nacional de Programas Preventivos y Control de Enfermedades), Secretaria de Salud, México City, México; 2 CENAPRECE, Secretaria de Salud, México City, México; 3 Global Health Research Group, School of Public Health and Community Medicine, Sahlgrenska Academy, University of Gothenburg, Gothenburg, Sweden; 4 Population Health Section, King Abdullah International Medical Research Center, Riyadh, Saudi Arabia; 5 Special Program for Research and Training in Tropical Diseases (TDR-WHO), World Health Organization, Geneva, Switzerland; 6 Centre for Medicine, and Society (ZMG), Freiburg University, Freiburg, Germany; Keele University, UNITED KINGDOM

## Abstract

Dengue disease epidemics have increased in time and space due to climatic and non-climatic factors such as urbanization. In the absence of an effective vaccine, preventing dengue outbreak relies on vector control activities. Employing computerized tools to predict outbreaks and respond in advance has great potential for improving dengue disease control. Evidence of integrating or implementing such applications into control programs and their impact are scarce, and endemic countries demand for experience sharing and know-how transfer. Mexico has extensive experience of pre-validated EWARS (Early Warning And Response System), a tool that was developed in 2012 as part of a collaboration with the Special Program for Research and Training in Tropical Diseases Unit (TDR) at the World Health Organization and used at national level. The advancement of EWARS since 2014 and its stepwise integration into the national surveillance system has increased the appreciation of the need for integrated surveillance (including disease, vector and climate surveillance), and for linking inter-institutional and trans-sectoral information for holistic epidemiological intelligence. The integration of the EWARS software into the national surveillance platform in Mexico was a remarkable milestone and a successful experience. This manuscript describes the implementation process of EWARS in Mexico, which started in 2012 and further demonstrates benefits, threats, and opportunities of integrating EWARS into existing national surveillance programs.

## Introduction

### The need for outbreak prediction

The increase of dengue in the last two decades from 500,000 cases in 2000 to 5.2 million in 2019 [1] and its spread outside the mere tropical countries since 2010, was also coupled with the introduction of outbreaks of Chikungunya (2013) and Zika (2014) in the Americas. The absence of effective vaccines and costly processes of surveillance systems has further augmented the need to reassess the current detection, prediction, and response approaches practiced for climate sensitive diseases. Current outbreak control measures are often perceived as suboptimal as they intervene late during the downward slope of disease outbreaks rather at the beginning of the epidemic curve. A practical framework was needed to anticipate the risk of disease transmission and to organize timely operations of control measures.

In line with the global and national disease control and response priorities, the Special Program for Research and Training on Tropical Diseases (TDR/WHO) convened in 2012 an expert meeting with ten Asian and American countries to discuss the possibility of developing an early warning system and response system (EWARS) for dengue outbreaks to guide a timely response for its control [2]. Mexico showed commitments to the proposed project of developing and piloting an EWARS tool for dengue outbreak prediction based on optimal statistical metric (sensitivity and positive predictive values) to predict in advance the onset of epidemic dengue transmission in a timely manner and with a systemized country-adapted stage-wise response and control.

**Fig 1:**
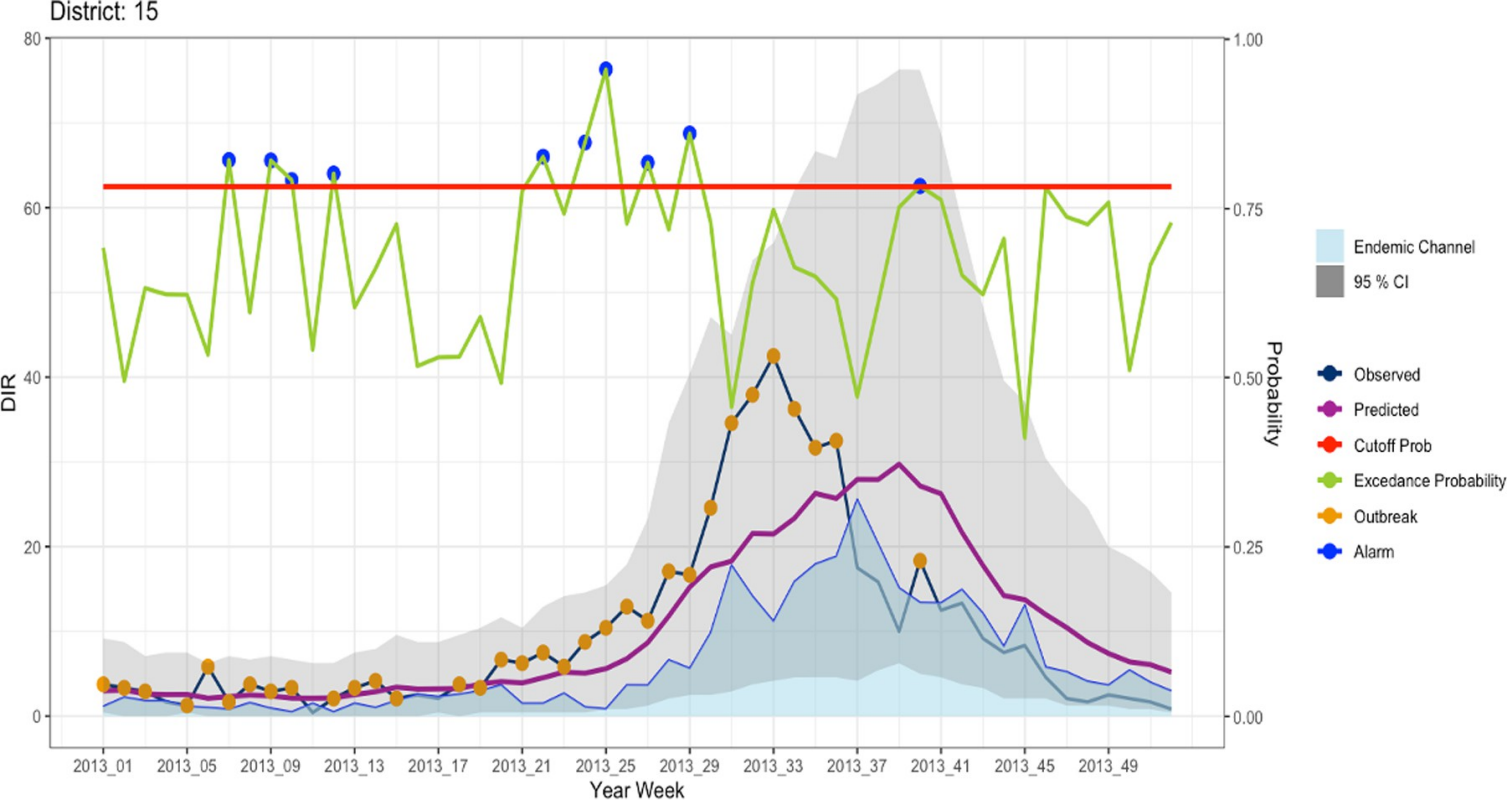
Overview of the model evaluation process, illustrating the endemic channel, observed and predicted hospitalized dengue cases, outbreak weeks (when number of cases exceeds the endemic channel), the generated probability of the number of cases to exceed the endemic channel and, the alarm signals which indicate when the probability of an outbreak crosses the cut-off, warranting for a possibly disease outbreak in the forthcoming period (here is 12 weeks ahead of the prediction week).

The statistical concept of EWARS is published elsewhere [3]. The model employs the robust Distribution Lag Non-Linear Model and combining it with the INLA Bayesian regression framework. The model produces out-of-sample predicted probabilities of exceeding the outbreak threshold. Weekly predictive probabilities of cases for each district (incidence rate) is first computed from alarm indicators parameters. As illustrated in Fig 1, this predicted disease probability is compared against thresholds (i.e. case number based on endemic channel) and the probability of exceeding the endemic channel (threshold) is calculated, and if this probability exceeds a calibrated cut-off value, alarm signals are then generated to warn of forthcoming disease outbreaks.

The updated EWARS ^**Plus**^ tool facilitates additional features to support unskilled users performing early descriptive measurements to ensure data reliability and usefulness for the prediction purposes. It also includes interactive graphical features to improve results interpretation for users at the national (central) and local (district) levels. The EWARS^**Plus**^ tool can comprehensively predict disease outbreaks in time and space and quantify the magnitude (outbreak rate) and its certainty interval, which will have significant vector control and response implications.

### The case of Mexico

Like many other dengue endemic countries, the epidemiological dengue surveillance in Mexico is based on the reporting of probable and/or laboratory-confirmed cases to the National Epidemiological Surveillance System, with a subsample of 30% sent for laboratory confirmation [2, 4–6]. Unlike the disease detection concept for establishing an outbreak reference line and response, which relies merely on the number of cases being reported, a set of relevant meteorological, entomological, or epidemiological alarm indicators are proposed to define a more advanced early warning process for generating predictive disease outbreak scenarios. By virtue of the biological association of meteorological, entomological and some epidemiological data with vector-borne diseases, such alarm indicators have shown evidence of predictive abilities of forthcoming disease outbreaks [7]. Meteorological data have dominated the overall predictive performance mainly if provided on time- and at area-specific level. Since the establishment of the Mexican entomological surveillance in 2008 [8], consistent weekly monitoring of ovitraps has been maintained, which has potential for multiple public health applications including the EWARS prediction process. With the support of the National Institute of Public Health, the "Comprehensive Vector Monitoring System, in the information subsystem for dengue surveillance" was launched, it is worth mentioning that this platform has been improved over the years for the benefit of the user (the operational and analytical personnel), which facilitated the opportunity of linking confirmed geo-referenced cases with ovitraps indicators such as the proportion of positive ovitraps and average number of *Aedes* eggs per block of houses. This led to the generation of the “Transmission Risk Indicator”, where the areas of greatest risk are those blocks (or clusters) with the highest number of vector eggs and the highest number of cases [9].

The transmission risk indicator made it possible to identify clusters towards which resources and control actions were targeted. Also targeted vector control measures were applied around probable cases (*focal spraying*). Recently, the identification of hot spots has been included in different operational scenarios for the control of *Aedes aegypti* and the transmission of dengue including other arboviruses [10]. The geographical area of initiating control activities is the municipality (referred to as “locality” in Mexico) but we will use the generic term of “municipality” in this paper, which also corresponds to a “health district”. In this article, the experience of the Mexican process of incorporating EWARS as part of the national comprehensive epidemiological surveillance of dengue will be reviewed and discussed.

## Nationwide implementation of EWARS in Mexico

### Overview of dengue surveillance platforms in Mexico

The vector control program in Mexico (CENAPRECE) operates its surveillance systems in parallel to the electronic platforms of the National Epidemiological Surveillance System (General Directorate of Epidemiology) and the Comprehensive Vector Monitoring System (National Institute of Public Health). While this verticality can facilitate the incorporation of new components into the information system programs, it may also complicate the construction of integrated platforms due to the typical bureaucracy and hierarchy processes in Mexico.

#### Creating a comprehensive electronic platform

One of the early challenges of the EWARS development, the processes of collection, piloting and validation of the data adopted by Mexico were hindered by the multiple changes suffered by the platform of the National Epidemiological Surveillance System in its Vector-Transmitted Diseases module and its collection tool, (change of operational definition, change of variables and change of laboratory algorithms) institutional necessary for the early warning system tool. The General Directorate of Epidemiology possesses and oversees an online database of geo-referenced dengue cases; the National Vector Program runs a comprehensive vector monitoring system of geo-coded weekly entomological information, and the National Meteorological Service provides weekly data on temperature, rainfall and relative humidity by municipality. While the three databases were managed independently, it was feasible to establish the institutional and inter-sectoral coordination, which connects and automatically processes the different data sets in a repository to generate the required indicators. Other alarm indicators included the percentage of positive laboratory tests, predominant dengue virus serotype and changes in the age groups of dengue patients. However, due to the limited weekly virus typing in local laboratories, it was difficult to receive weekly information on serotype and seropositivity. In addition, the introduction of new serotypes as well as the variation of patients’ age groups could be suitable indicators for regional (State) and national level not for the local (municipality) level due to lower case numbers.

The geo-referencing of cases assigned the municipality (“locality”) as the spatial unit of data reporting and processing. The entomological surveillance system, which is based on ovitraps monitoring [11, 12], additionally incorporated entomological information to correlate case numbers with estimated vector densities on a weekly basis and per locality. Weekly hospitalized dengue cases are the best indicators to define dengue outbreaks [13] and were proposed for this EWARS purpose in Mexico. Subsequently, probable or lab-based cases were used for outbreak prediction when low hospitalization rates were observed in some areas [14].

### The development and validation of EWARS prediction algorithms using retrospective data

In 2014, five out of ten WHO-partner countries, with strong surveillance systems (Mexico, Brazil, Dominican Republic, Malaysia and Vietnam) participated in this second stage of the EWARS development process. The utilized retrospective (historical) data covered the period of 2007 to 2011, which defined the run-in period particularly responsible for developing area-specific (municipality) prediction algorithms. The second part of the historical data defined the evaluation period, which covered the years 2012 to 2013 to test the model sensitivity and positive predictive values of outbreak prediction using multiple alarm indicators. In the case of Mexico, the variables of weekly information that were used for the prediction process included 1) epidemiological alarm indicators; ‘patients’ mean age’, ‘circulating serotype’, 2) entomological alarm indicators; ‘percentage of positivity’ in laboratory tests, ‘average of positive ovitraps’ and ‘average of vector eggs per block’ and, 3) meteorological alarm indicators; ‘mean temperature’, ‘relative humidity’ and ‘total weekly rainfall’. Both ‘probable’ and ‘hospitalized’ dengue cases were used as the outbreak indicator to investigate optimal predictors [13].

Mexico was one of the three countries where it was possible to validate the statistical algorithm performance due to the availability of complete and consistent data sets. The results of the retrospective study met the objective of identifying the most useful ‘predictive and ‘outbreak’ variables and essentially managed to evaluate the highest sensitivity and predictive values in different calibration and prediction scenarios by testing the performance of the computer assisted prototype against different thresholds. The analysis confirmed the quality of the epidemiological data and the importance of continuing to manage ‘probable’ and ‘hospitalized’ dengue cases as valid outbreak variables, with preference towards hospitalized cases. The Mexican General Directorate of Epidemiology has thoroughly reviewed the historical database and carried out the incorporation of complete and clean weekly retrospective data prior to the annual statistical closure. However, it is important to recognize that on a day-to-day basis, health services make decisions based on the available data, despite the fact that these data have a delay of two weeks on average. This delay is independent of the tool’s prediction performance measured by the sensitivity and predictive value as the calibration process of the prediction model relies on retrospective “clean” data while the prospective part of outbreak prediction utilizes climate and other predictors, which are presumed available on a weekly basis.

The unavailability of weekly alarm predictors data limited the inclusion of ‘percentage of positive serological test’ despite those countries had a consensus that ‘positivity in laboratory diagnostic tests’ could be one of the most robust alarm indicators. Another example was the use of entomological indicators; those indicators are often collected randomly without defined periodicity, which prohibits their applications for outbreak prediction. Out of the partner countries that participated in the retrospective study, only the Mexican ovitrap system fulfilled the requirements of an entomological alarm indicator. It is worth mentioning that the indicator “proportion of positive ovitraps” had a higher sensitivity as alarm indicator than “number of eggs per-block”.

### Building prospective evidence-based response using EWARS

In order to complement the assessment of the EWARS implementation process, CENAPRECE in coordination with TDR, validated EWARS for dengue outbreaks in 2017 [15] across 11 of the 20 municipalities of the prospective study. The results demonstrated that districts with adequate and timely response guided by alarm signals—graphical or numerical notifications generated by the prediction model when the estimated probability of an upcoming outbreak exceeds the defined outbreak threshold in a particular district—demonstrated successful records of outbreak prevention. The study noted the different levels of compliance with response protocols, highlighting the importance of training and availability of resources to standardize local response capacity. This provided additional motivation to scaling up the process of incorporating EWARS at the national system of Integrated Epidemiological Dengue Surveillance in Mexico.

### Integrated epidemiological surveillance of Dengue and EWARS

Based on the results of the above presented studies and the evaluations of EWARS as well as the timely response to control dengue outbreaks from 2012 to 2017, Mexico decided in 2018 to incorporate EWARS into the national platform for integrated epidemiological surveillance of dengue.

Prior to the integration of EWARS, a set of challenges were predominantly identified and addressed early in time to pave the way for a more adequate EWARS integration process. Overall, while EWARS is technically designed to be aligned with an already existing routine mechanism of disease warning and response process, there was a heterogeneous state of acceptability among users at both the central (MOH) and local (municipality) levels. The quality of data representation, completeness and reporting mechanism remains a challenge, mainly different diseases have different surveillance scenarios and meteorological data are essentially provided via transitory stations, often with broad geographical areas which reduces the statistical prediction performance of EWARS. Initially, the epidemiological, entomological, and vector control data were available but operated via independent database servers that operated on independent servers, which distracted the timely data feeding and internal communication channel. The meteorological data were only partially available on independent databases and both the General Directorate of Epidemiology, and the Vector Control Program had access to the complete information only in a semi-automatic manner. Prior to the integration of the CENAPRECE, it was necessary to download the databases and process them manually before being used, a process that would typically demand a mutual agreement on a common repository where databases can be automatically intertwined to generate needed information for feeding EWARS. During this integration work period, COVID-19 has dominated all staff attention which impacted the surveillance of vector-borne diseases and thus the EWARS application. The sustainability process of EWARS was therefore an important piece among the list of challenges. Nevertheless, the IT sector in CENAPRECE is well established, which secured a national platform (server) for hosting, modifying and managing the EWARS package, which is a crucial aspect in this overall EWARS integration puzzle.

To harmonize the data challenges, mainly in relation to the meteorological data, it was essential to include additional important stakeholders that are the National Water Commission and its National Meteorological Service. However, as there were no fixed meteorological stations in all municipalities, which was coupled with difficulties to continue acquiring portable meteorological stations, it was decided to include a climatologist in the team. Key recommendations were mutually proposed by the climatology expertise; 1) To adopt the weather prediction model of the National Oceanic and Atmospheric Administration (NOAA) applying a programming component for automating data, which has complete records since 1973 and incorporate it into EWARS. 2) To create an interface to multiple software packages normally distributed under a free- or open-source license; 3) To install climate data operators to handle and analyze data from climate models and numerical weather prediction models using the common network data formats developed by the Max Planck Institute for Meteorology [16] and, 4) To employ a programming language compatible with "R", the operative language of EWARS, for auto-feeding climatic data in a timely prospective manner.

**Fig 2:**
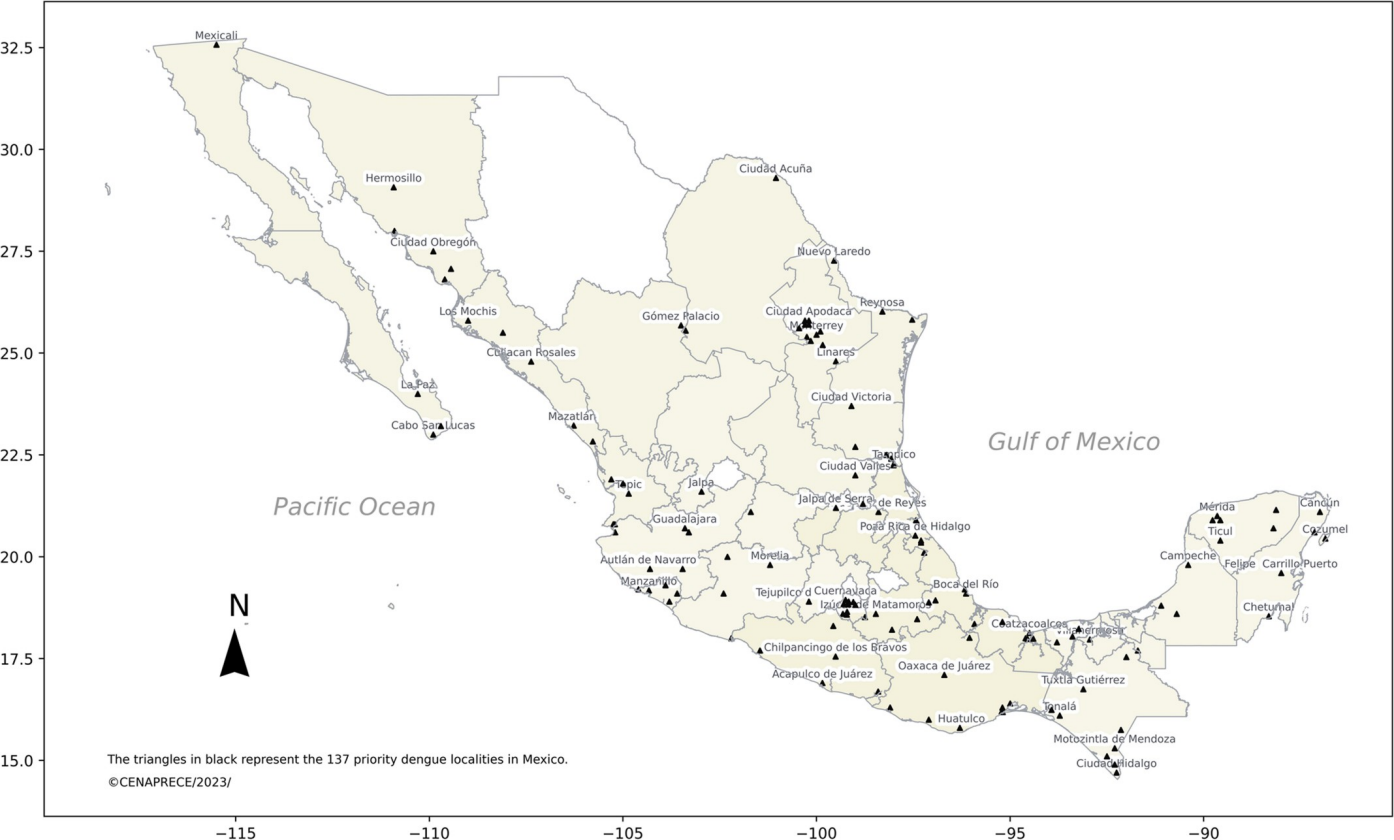
Map of the 137 priority dengue localities (sentinel) where EWARS is implemented, Mexico 2023. The shapefile of the political divisions of Mexico belongs to INEGI 2022 edition, with a scale of 1:4000000 available online and freely accessible. (https://www.inegi.org.mx/temas/mg/).

Once the data by municipality (location) was downloaded, the geo-coordinates were tagged and verified. The country was divided into eight sub-regions for greater power of resolution, the closest nodes were interpolated with respect to the coordinates of interest. Resolution changed to 5X5 km; and the data series were extracted daily and stored to prepare national maps for temperature, rainfall and relative humidity. The results of outbreak prediction are published on a weekly basis for 137 priority municipalities (localities) and supervised by the central level at CENAPRECE thus augmenting the communication between local- and central-levels (Fig 2). Recommendations are given by EWARS as a reminder, of which a timely response according to the national protocol should be activated when outbreak alarms are noticed.

The acquisition and installation of transitory meteorological data was carried out between 2018 and 2019, a period when a new government administration came into office, with new decisions regarding health information systems and epidemiological intelligence. It was, however, agreed in 2019 to continue the work for the formalization of integrated epidemiological surveillance, including EWARS. Unfortunately, the COVID-19 pandemic attracted the full attention of the Health Services, mainly for epidemiological surveillance, delaying the final completion of the EWARS process.

### Innovating the EWARS process: Modernizing the integration of EWARS

*Findings* from a fairly recent retrospective study [15] in Mexico and Colombia have demonstrated a capacity of EWARS for the prediction of dengue (and Zika) outbreaks with a sensitivity of 97%, and positive predictive values of 68%. The prediction period between the alarm and the start of the outbreak was 6 to 8 weeks. This would operationally allow adequate and step-wise responses in controlling outbreaks, reducing serious cases and deaths compared to when the alarm comes only after an increase of cases (outbreak detection).

The use of the open access “R” software in EWARS ^plus^, the automation of its operation, the ease of its interpretation and its usefulness for multiple climate-sensitive diseases (dengue, Zika, chikungunya, Malaria, cholera, diarrheal diseases) as well as the further development of its software has augmented the tool applications with improved response in time and space. The machine learning-based EWARS ^plus^ is of a user-friendly use and interpretation design capable of not only to general early spatial-temporal signals but to predict disease incidences (i.e., the magnitude of the outbreak) and their confidence intervals, which can have further significant vector control and response implications. Despite being cheap and popular, applying the open-access R package is associated with sophisticated and complex applications for routine implementation work. A docker image approach–which can virtually store and execute all related packages and applications associated with the EWARS installation—has more recently been achieved to simplify the EWARS integration process within national surveillance programs and other key entities. This was made so flexible that users with local (data-based) or web-based (cloud) servers can both benefit from the docker image approach.

## Discussion and recommendations

The EWARS program–developed by TDR with partner institutions and partner countries–has been introduced in Mexico since over 10 years as a step-by-step process illustrating phases of monitoring and evaluation, as well as further development and documentation of prospects and limitations. It has demonstrated its usefulness for the prediction of climate sensitive disease outbreaks mainly to trigger and guide early responses aiming at the mitigation or prevention of outbreaks. However, capacity building for the appropriate management of EWARS as well as for the generation and processing of high-quality surveillance data is needed to offer the appropriate and timely response.

The case of Mexico can provide some key recommendations to ensure successful and meaningful use of EWARS; while EWARS is a resource with potentials to provide greater benefits, it should be locally owned and adapted to the conditions and resources available in the country. It would be crucial to engage all key stakeholders at early stage of the implementation process. This does not only relate to the national disease control program, but also to the local meteorological entity, the IT expertise and essentially prioritizing a local server to host and link EWARS to different sentinel sites at national levels. While meteorological data appear to be best outbreak predictors, such information are often aggregated at large geo-scale reducing their predictive usefulness. Hence, seeking satellite data has great potential of improving the prediction quality. Automating the in- and out-data feeding between the surveillance and meteorological databases can ensure sustainability of the tool and improves consistency of the process.

EWARS is perceived as a coordinating tool improved dengue surveillance, prevention, and enabling efficient control programs to be increasingly anticipatory and less reactive when implementing control measures. This has been particularly illustrated in the Mexican example, where high technical competence (automatization of data handling, interface of different data platforms, use of meteorological intelligence), close collaboration of different government departments (epidemiology and vector control) and of different sectors (health, meteorology) have made optimal use of the EWARS tool developed by TDR together with other institutional partners (University of Freiburg, Germany and University of Gothenburg, Sweden) and endemic countries in Latin America, Asia and Africa. Currently, more than 17 countries either exercising, fully implemented or in the process of integrating EWARS within national programs with efforts to share similar experience of the process and further recommendations. While this paper has focused on the case of dengue in Mexico, the tool has been tested for Chikungunya Fever, Malaria, Cholera and Zika Virus Disease and has potential for meningitis and other climate-sensitive diseases where predictive data are available.
